# Plasma and Urinary Amino Acid-Derived Catabolites as Potential Biomarkers of Protein and Amino Acid Deficiency in Rats

**DOI:** 10.3390/nu13051567

**Published:** 2021-05-07

**Authors:** Joanna Moro, Nadezda Khodorova, Daniel Tomé, Claire Gaudichon, Catherine Tardivel, Thierry Berton, Jean-Charles Martin, Dalila Azzout-Marniche, Delphine Jouan-Rimbaud Bouveresse

**Affiliations:** 1 UMR PNCA, AgroParisTech, INRA, Université Paris-Saclay, 75005 Paris, France; joanna.moro@agroparistech.fr (J.M.); nadezda.khodorova@agroparistech.fr (N.K.); tome@agroparistech.fr (D.T.); claire.gaudichon@agroparistech.fr (C.G.); delphine.bouveresse@agroparistech.fr (D.J.-R.B.); 2 UMR C2VN, Aix-Marseille Université, INSERM, INRAE, 13385 Marseille, France; c.tardivel@univ-amu.fr (C.T.); thierry.berton@univ-amu.fr (T.B.); jean-charles.martin@univ-amu.fr (J.-C.M.)

**Keywords:** protein, amino acid, requirement, deficiency, metabolomics, biomarkers

## Abstract

Objective: Dietary intakes must cover protein and essential amino acid (EAA) requirements. For this purpose, different methods have been developed such as the nitrogen balance method, factorial method, or AA tracer studies. However, these methods are either invasive or imprecise, and the Food and Agriculture Organization of the United Nations (FAO, 2013) recommends new methods and, in particular, metabolomics. The aim of this study is to determine total protein/EAA requirement in the plasma and urine of growing rats. Methods: 36 weanling rats were fed with diets containing 3, 5, 8, 12, 15, and 20% protein for 3 weeks. During experimentation, urine was collected using metabolic cages, and blood from the portal vein and vena was taken at the end of the experiment. Metabolomics analyses were performed using LC-MS, and the data were analyzed with a multivariate analysis model, partial least Squares (PLS) regression, and independent component-discriminant analysis (ICDA). Each discriminant metabolite identified by PLS or ICDA was tested by one-way ANOVA to evaluate the effect of diet. Results: PLS and ICDA allowed us to identify discriminating metabolites between different diet groups. Protein deficiency led to an increase in the AA catabolism enzyme systems inducing the production of breakdown metabolites in the plasma and urine. Conclusion: These results indicate that metabolites are specific for the state of EAA deficiency and sufficiency. Some types of biomarkers such as AA degradation metabolites appear to be specific candidates for protein/EAA requirement.

## 1. Introduction

The quality and the quantity of protein intake has become a particularly sensitive issue in the context of current debates on both the increase in the world population, the problem of child undernutrition, and the need to rebalance animal and vegetable food sources. Food proteins provide the amino acids (AA) necessary for the synthesis of proteins and various nitrogenous molecules in the body. Proteins are major structural constituents of tissues, having a role in structure or with biological functions with an immune role or as an enzyme, hormone, transporter, or receptor [1]. In healthy adult humans, the body protein compartment is estimated to be 15% of the individual’s weight. Every day there is a renewal of the protein pool by proteolysis and proteosynthesis estimated at 250–300 g per day (2% on average) [1].

The maintenance of tissues and physiological functions in adults and the support of growth in the young require a balance of the body free AA pool, renewed by food intake and body proteolysis. In humans, 20 AA are called “proteinogenic”, and among these, 9AA are considered essential (EAA), because their carbon skeleton is not or poorly synthesized in the body and they must therefore be provided by food. Protein synthesis depends critically on a balanced intake of dietary AAs, and particularly EAA, as protein can only be synthesized if all the constituting AAs are simultaneously available in adequate quantity. The ability to supply EAAs at a level sufficient to meet nutritional needs is critical and requires precise data on the needs in each EAA.

Various methods for measuring EAA requirements have been discussed for decades. First, proteins (from AA) are the main nitrogen-containing compounds in the diet and the body. Thus, the body need can be seen as the nitrogen retention of the intakes, and the needs were assessed using the nitrogen balance method. This method consists of identifying the difference between nitrogen inputs and losses (urinary, fecal, and others) [2,3]. However, this method presents some limits to determining EAA requirements because of the underestimation of losses, overestimation of intake, and a low number of subjects in each study [4]. Alternative methods have therefore been proposed using isotopic tracers. The first is the direct AA oxidation method, based on the carbon-13 labeled AA infusion and on the measure of oxidative losses in the form of CO_2_ during the fasting and fed states [5]. The second is the indicator AA oxidation method, measuring the oxidation of indicator AA, usually phenylalanine, depending on the level of intake of EAA studied [5,6]. However, these different methods provide a wide range of EAA requirement values. In the case of lysine, the requirement estimated with the indicator AA oxidation method is almost four times higher than that estimated with the nitrogen balance (45 mg/kg/d vs. 12 mg/kg/d) [7], and this wide range does not allow for the determination of inter-individual variability and therefore for defining nutritional recommendations.

The FAO recommends considering EAA as individual nutrients and has established different characteristics to develop new methods. According to these characteristics, methods must be minimally invasive, directly applicable to humans in different physio-pathological conditions, and applicable to each EAA with a reasonable cost [8]. The emergence of new technologies and “omics” sciences, such as metabolomics, seem to hold promise for developing a new method of measuring the requirement for AA.

The aim of this study was to determine biomarkers of EAA in plasma and urine and protein sufficiency using a model of growing rats fed with a low protein/AA diet for three weeks. The hypothesis was that AA deficiency should induce an increase in the enzymatic systems of catabolism of other AA with the production of degradation metabolites in biological fluids that can disappear when the protein intake reached the requirement, and thus these metabolites can be used as a biomarker of sufficiency.

## 2. Materials and Methods

### 2.1. Animals

The study was approved by the Regional Animal Care and Ethical Committee and the Minister of Research and conformed to the European legislation on the use of laboratory animals (registration number: APAFIS#13436-2017122616504600). Thirty-six male Wistar Han rats (HsdHan^®^: WIST, Envigo, France), aged 3 weeks and weighing 50–55 g on arrival, were housed individually in the light- and temperature-controlled animal facility of AgroParisTech (12-h light/12-h dark cycle, lights on from 00:00 to 12:00, 22 °C). Rats were fed with a standard rat chow diet (Régime croquettes from Safe, 16.10% of protein) during one week of adaptation before being switched to their experimental diet for three weeks.

### 2.2. Pellet Preparation

Food pellets were prepared in the laboratory using powder manufactured at the “Atelier de préparation des aliments” (Sciences de l’Animal et de l’Aliment de Jouy (SAAJ), INRAe, Jouy en Josas, France). Powder and water were mixed to form a dough to prevent powder crumbling and to measure food intake. This dough was cut into pellets and left to dry for 3 days before being given to the rats. During these 3 days, the progressive loss of water was calculated to adjust the dry weight of the pellets.

### 2.3. Experimental Design

After one week of adaptation to the laboratory conditions, the thirty-six rats were divided into six groups (*n* = 6/group) and were assigned one of the 6 isocaloric diets containing respectively 3, 5, 8, 12, 15, or 20% milk protein, respectively, noted as P3, P5, P8, P12, P15, and P20, respectively, where P stands for protein (Table 1 and Table S1). Each day for 3 weeks, a calibrated meal of 4 g (58.2 kJ) was given at 12:00am (onset of the night period). This calibrated meal is given to train the rats to rapidly ingest a small amount of food. Ad libitum access to food and water was given between 12:30am and 9:00am the next day. Food intake and body weight were measured daily during the three weeks. In the 3rd week, each rat was placed for 48 h in metabolic cage (24 h of adaptation and 24 h of measurement) to collect urine for metabolomics analysis.

At the end of the study, at 11:00am, the rats were fed with a 4-g pellet (58.2 kJ) of their test diet to standardize the energy intake, and two hours later, they were anesthetized with Isoflurane. Blood was taken from the portal vein and from the vena cava. The blood samples were collected on heparin, centrifuged (4 °C, 3000 rpm, 10 min), and plasma was stored at −80 °C until metabolomics analysis. Thereafter, body composition was analyzed by dissection and the weighing of the main tissues and organs.

### 2.4. Urine Sampling

During 24 h of measurement, rats were housed individually in metabolic cages. These metabolic cages have a perforated bottom for the collection and separation of urine and feces. Feces and urine pass through a collection funnel and are both separated and collected in two different tubes. The cages are equipped with feeding bowls and bottles, and the rats were fed ad libitum with their respective diets.

### 2.5. Metabolomics Sample Preparation and Liquid-Chromatography Mass Spectrometry (LC-MS) Analysis of Urine and Plasma

Plasma and urine samples were analyzed as described [9] on two different columns in LC-HRMS: C18 and HILIC. In order to broaden metabolite coverage, the acquisition was operated both in positive and negative ionization mode for each chromatographic condition. Four result matrices were therefore obtained before being merged. Metabolite redundancy was removed by choosing variables with less coefficient of variation (CV) and higher intensity in quality control samples. The following workflow was applied to all samples: on the C18 column, during a 16 min gradient, samples were analyzed with solvents A and B, 0.1% formic acid in H_2_O, and 0.1% formic acid in acetonitrile, respectively; on the HILIC column, samples were analyzed during a 27 min gradient with solvents A and B, 16 mM ammonium formate in H_2_O, and 0.1% formic acid in acetonitrile, respectively. All samples were analyzed in FullScan and some were also analyzed in MS/MS to facilitate the annotation of compounds. Every 5 samples, a pool of all samples (quality control sample, QC) was injected to correct instrumental variation during the assay sequence. Data extraction was made by XCMS from raw data acquired in FullScan. Data were curated by visual inspection of EIC (extracted ion chromatogram) to remove extracted chemical noise and poorly integrated peaks. Data were normalized to QC injected every 5 samples with the Van Der Kloet algorithm using R scripts, in order to correct instrument analytical drift. Variables with coefficients of variation greater than 30% in the pools were removed. Finally, the selected variables were matched with the internal database of the laboratory containing over 1000 pure metabolites with their mass spectra and retention time using the data in-house LCMS tool of the W4M platform (https://workflow4metabolomics.org/).

### 2.6. LC-MS Data Processing and Analysis

The collected data were separated into three data sets, containing the mass spectra of (1) the urine samples (Urine data set), (2) the portal vein samples (PV data set), and (3) the vena cava data set (VC data set), and 3 matrices, of respective dimensions 36 × 245, 36 × 141, and 36 × 141 were obtained. They were first pretreated before applying multivariate analysis methods.

#### 2.6.1. Data Pretreatment

The mass spectra were first pretreated with the probabilistic quotient normalization method (PQN) [10] using the median signal as a reference vector. PQN is a normalization method enabling the reduction of uncontrolled variations due to instrumental factors in the signals. Pareto scaling [11] was then applied to the normalized data. Pareto scaling is a pretreatment method where the intensity of each centered *m*/*z* variable is divided by the square root of its standard deviation, which enables it to give more weight to small-intensity variables which are potentially significant. Multivariate analysis methods were then applied to the pretreated data.

#### 2.6.2. Development of a Multivariate Model

The chemometrical data analysis carried out in metabolomics is usually based on qualitative models, i.e., one wants to check whether the presence or absence of a nutriment, for example, has an influence on the metabolome. This can be analyzed either by unsupervised methods, such as principal components analysis (PCA) [12], or by supervised methods, such as partial least squares for discriminant analysis (PLS-DA) [13]. However, given the range of ingested protein concentrations available in the present study, it was interesting to check whether a pertinent quantitative model between the mass spectra and the corresponding concentrations could be obtained. Therefore, both a quantitative and a qualitative model were calculated. The quantitative model used in this study was partial least squares (PLS) regression [14]. As for the qualitative model, a novel method was developed recently, which consists of a supervised version of independent component analysis (ICA) [15], called independent component-discriminant analysis (ICDA) [16]. It has been shown to perform at least as well as PLS-DA, but has the additional advantage of simplifying the interpretation of the obtained factors, and was hence used in this study.

##### Partial Least Square (PLS) Regression

PLS is a multivariate quantitative analysis, which relates one vector, denoted as y (here, the concentration of ingested proteins) to a matrix denoted as X (here the mass spectra) by calculating the parameters of a linear model. The idea underlying the use of a quantitative method in this metabolomic study is as follows: one wants to check whether a low-protein diet has an impact on the metabolome. Since different concentrations of proteins are available, this influence, if it exists, is likely to be dependent on the amount of ingested proteins. This can be checked by applying a quantitative model such as PLS. The calculation of a PLS model implies the determination of new variables, called latent variables (LVs), which are linear combinations of the original variables (the pretreated *m*/*z*), having several interesting properties: (1) they are orthogonal, and (2) they are sorted in decreasing amount of covariance between X and Y. Therefore, the first A LVs contain systematic variation due to information relating the two matrices, and are important for the model, while the latter LVs are related to random variation due to noise and should not be included in the model. One important factor in the construction of a PLS model is the determination of the number A of LVs to include in the model. In the present study, due to the relatively small number of analyzed samples, this was achieved by cross-validation. Once the optimal number of LVs is obtained, the final model can be constructed. The regression coefficients can then be used as indicators of the original variables importance.

##### Independent Component Discriminant Analysis (ICDA)

**Figure 1:**
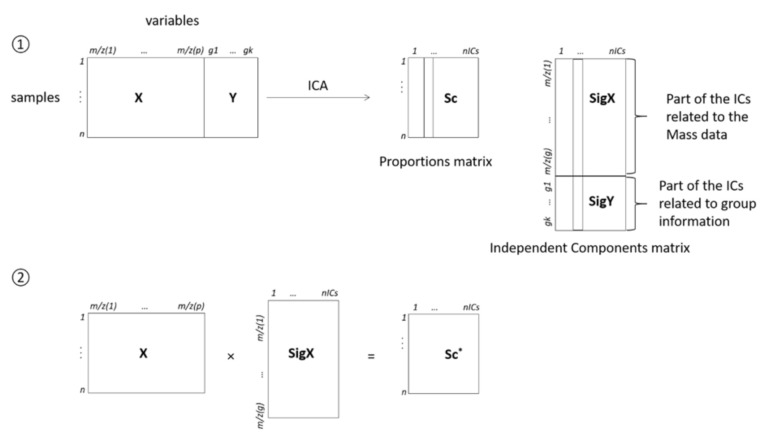
Schematic representation of ICDA. X is the matrix of mass spectra and Y is the group information matrix (there are as many columns as groups, and for each group, there is a 0 value if the corresponding sample does not belong to this group, and a 1 if it does). Sc is the proportion matrix, while SigX and SigY correspond to the parts of the signal matrix related to the mass spectra and to the group membership, respectively; Sc * is the proportion matrix obtained after removing SigY from the calculations.

ICDA [16] is a method derived from independent components analysis (ICA). In order to apply ICA, one has to assume that the experimental data are equal to a linear combination of pure component signals. With only the experimental data at hand, ICA aims at finding both the pure sources as well as the proportions of these sources in each of the experimental signals. The pure components are obtained in a so-called “signal matrix” (equivalent to the loadings matrix in PCA), while the proportions are obtained in a “proportions matrix” (equivalent to the scores matrix in PCA). The ICDA method combines the power of ICA to obtain pure interpretable components with the inclusion of information about the samples (group membership, i.e., indication of the group to which each sample belongs) in the model in order to orient the ICs towards discrimination. This method has been successfully used in a number of studies [16,17]. Here, it has been applied by splitting the samples into 3 groups: (1) very low protein concentrations (P3 and P5); (2) low protein concentrations (P8 and P12); and (3) normal protein concentrations (P15 and P20). The use of three groups rather than 6 groups (one per concentration) has been made after a careful data inspection, where it could be seen that 3 groups were likely to yield a better discrimination than 6 groups. A schematic presentation of ICDA is presented in Figure 1.

In the first step, the X and Y matrices are concatenated, and an ICA model is calculated on the obtained matrix. As explained earlier, ICA yields two matrices, namely a proportion matrix (Sc in Figure 1), which indicates the proportions in which each pure source signal is present in the considered experimental signal, and a signal matrix (Sig), which, in the case of ICDA, is equal to the concatenation of two different signal matrices related to the mass spectra (SigX) and to the group membership matrix (SigY), respectively. Investigation of the proportions of each IC can help in detecting the ICs that are discriminating one group versus the others. Investigation of the variables with high intensities (in absolute value) on a discriminating IC in SigX helps in detecting the important original (*m*/z) variables. Investigation of the variables with a high intensity in SigY helps to find out which group is discriminated against by the corresponding IC, which can also be seen on the proportion plots. Yet, these proportions have been calculated by using both the information from X and from Y, and it is highly probable that the information in Y enhances the observed discrimination. Therefore, in step 2, new proportions are calculated (Sc *) by projecting the X matrix only on the SigX signals, i.e., by using only the information from the mass spectra. The new proportions obtained are more realistic; they can be plotted, and if a discrimination can still be observed, it means that there is information in the (urine or plasma) samples which is related to the amount of ingested proteins.

#### 2.6.3. Analysis by Univariate ANOVA Model for Discriminant Metabolites

Statistically, LC-HRMS matrices were analyzed, firstly, with multivariate methods such as ICDA and PLS in order to determine a set of discriminant molecules. In order to determine if some biomarkers could signify AA deficiency on their own, we performed a second univariate statistical test, an ANOVA. This test was used on each discriminant molecule found with multivariate methods to see if there is a difference between groups and if these molecules could act as a marker of AA deficiency. For each discriminant metabolite identified by PLS or ICDA, the effects of the diets were tested by one-way ANOVA using R^®^ software. Pairwise comparisons were performed with post hoc Bonferonni tests for multiple comparisons. Differences were considered significant at *p* < 0.05.

## 3. Results

Plasma (portal vein and vena cava) and urine samples were analyzed using LC-HRMS, and the MS data obtained were pretreated and analyzed using the PLS and ICDA methods.

### 3.1. Urinary and Plasma Biomarkers Obtained with PLS Method

**Figure 2:**
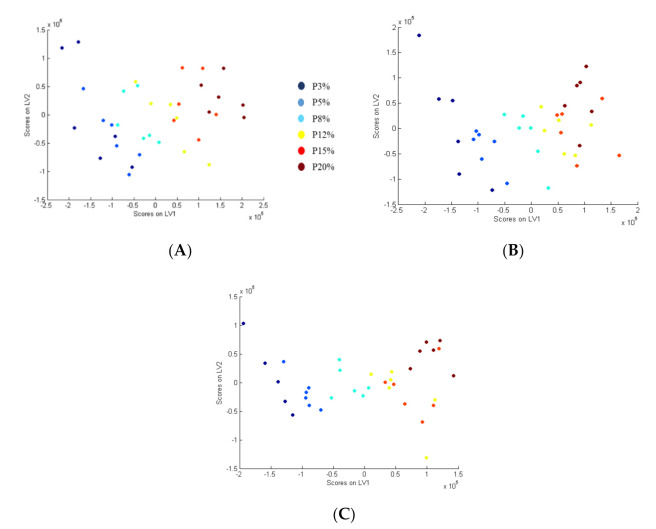
PLS Analysis for the data obtained by LC-MS characterizing the discrimination of the predefined groups to discriminate protein content diet based on IC1 and IC2 in urine (**A**), portal vein (**B**), and vena cava (**C**).

In the urine, portal vein, and vena cava samples, the six protein groups (P3, P5, P8, P12, P15, P20) were separated along LV1 (Figure 2). The S-Plot and QQ-plot were used to select the most relevant variables for the separation of the groups. The selection of discriminant variables was based on correlations and covariance in the S-plots and contributions to the LV that were greater than +/− 1, 2, and 3 standard deviation of all the contributions to each component in the QQ-plots.

For urine samples, PLS method revealed that forty-two features were associated with the consumed diets (Table 2 and Figure S1). With an ANOVA test, twenty-four molecules had different intensities according to groups. For eleven molecules the intensity was inversely related to protein in the diet, namely 3-methyl-2-oxovalerate, 4-methyl-2-oxovaleric acid, azelate, creatine, d-mannose, d-raffinose, galactose, phosphoric acid, pyridoxine, raffinose, and succinic acid. For twelve molecules, the intensity was positively related to protein in the diet, namely anthranilate, cadaverine, homogentisic acid, isovaleroylglycine, kynurenic acid, l-gulonolactone, *n*-methyl-2-pyridone-5carboxamide, pipecolate, uracil, xanthurenate indoxyl sulfate and putrescine. One other compound was group specific, namely pantothenate, as its concentration is not linear and does not depend on the percentage of protein. This molecule is decreased from P5 to P20, but P3 is not different from P12, P15, or P20.

For portal vein plasma samples, nineteen discriminant molecules were observed between groups. After the ANOVA test, sixteen variables were significantly different between the tested groups (Table 3 and Figure S1). Among these molecules, eight decreased in intensity when the percentage of protein intake increased, namely betaine, fucose, lysoPC (18:0), l-carnitine, l-histidine, l-serine, malate, and *o*-acetyl-carnitine, while eight increased in intensity when protein in diet increased, namely l-methionine, l-phenylalanine, l-threonine, l-tyrosine, *n*-methyl-2-pyridone-5-carboxamide, taurocholic acid, taurocholic acid, and tryptophan.

For vena cava plasma, twenty-two discriminant molecules were observed between groups. Fifteen molecules were statistically significant after ANOVA test (Table 3 and Figure S1). Eight molecules decreased in intensity when protein intake increased, namely 2-hydroxyisocaproic acid, betaine, fucose, lysoPC (18:0), l-histidine, l-ornithine, l-serine, and malate. On the contrary, seven other molecules increased in intensity when protein intake increased, i.e., 3-isopropylmalic acid, l-lysine, l-methionine, l-phenylalanine, l-threonine, l-tyrosine, and *n*-methyl-2-pyridone-5-carboxamide.

### 3.2. Urinary and Plasma Biomarkers Obtained with ICDA Method

**Figure 3:**
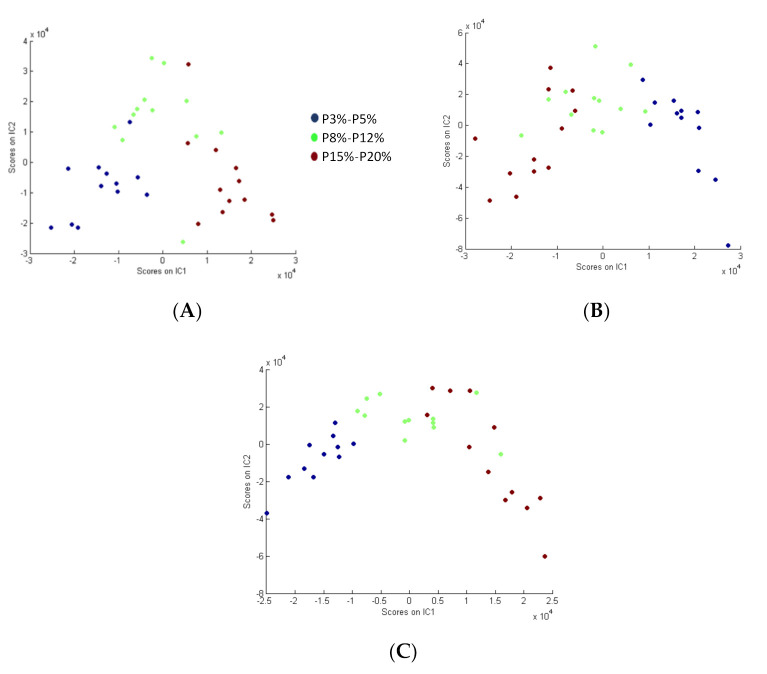
Independent components-discriminate analysis proportions plot calculated after source signal extraction for Table 1. IC2 in urine (**A**), portal vein (**B**), and vena cava (**C**).

In urine and portal vein and vena cava plasma, three different diet groups (very low protein, 3% and 5%; low protein, 8% and 12%, and normal protein diet, 15 and 20%) were separated along IC1 and IC2 (Figure 3) after source signal extraction by ICDA based on three groups from the data obtained by LC-HRMS. Similarly to what was done after PLS modelling, the S-Plot and QQ-plot were used to select the most relevant variables for the separation of the groups. The selection of discriminant variables was based on correlations and covariance on S-plots, and on contributions to the IC that were greater than +/− 1, 2, and 3 standard deviation of all the contributions to each component in the QQ-plots.

For urine samples, the ICDA method revealed that fifty-four features were associated with the consumed diets. After an ANOVA test, a total of thirty-five variables were considered statistically different between groups (Table 2 and Figure S2). Among these variables, sixteen metabolites, namely, 4-pyridoxate, anthranilate, cadaverine, homogentisic acid, indoxyl sulfate, isovaleoylglycine, kynurenic acid, l-gulonolactone, l-leucine, *n*-acetylputrescine, *n*-methyl-2-pyridone-5-carboxamide, pipecolate, putrescine, spermidine, uracil, and xanthurenate, were up-regulated when the percentage of protein increased. Eighteen metabolites, namely, 3-methyl-2-oxovalerate, 4-methyl-2-oxovaleric acid, alpha d-glucose, azelate, d-mannose, d-raffinose, galactitol, galactose, *iso*-maltose, l-carnitine, pantothenate, phosphoric acid, proline-leucine, pyridoxine, raffinose, succinic acid, sucrose, and creatine, were down regulated when protein in the diet increased. One metabolite, tyramine, was group specific, and decreased in P3 and P5, increased in P8 and P12, and again declined in P15 and P20.

For the portal vein plasma, thirty variables were found significant between groups with ICDA analysis. After an ANOVA test, twenty-one variables were discriminant (Table 3 and Figure S2). Eleven metabolites increased in intensity when the percentage of protein in the diet increased, namely, indoxyl sulfate, l-lysine, l-methionine, l-phenylalanine, l-threonine, l-tyrosine, *n*-methyl-2-pyridone-5-carboxamide, taurocholic acid, tryptophan, and l-valine. Eleven other metabolites decreased in intensity when the protein in the diet decreased, namely, betaine, fucose, lysoPC (18:0), l-carnitine, l-histidine, l-serine, *o*-acetyl-carnitine, pyroglutamate, l-ornithine, l-arginine, and citrulline.

Finally, for the vena cava plasma, thirty-four variables were revealed by ICDA. After an ANOVA, eighteen molecules remained statistically different between groups (Table 3 and Figure S2). On the one hand, nine variables increased in intensity when the percentage of protein in the diet increased, namely, l-leucine, l-lysine, l-methionine, l-phenylalanine, l-threonine, l-tyrosine, *n*-acetylserotonin, *n*-methyl-2-pyridone-5-carboxamide, and tryptophan. On the other hand, ten variables decreased in intensity when the protein in the diet decreased, namely betaine, fucose, lysoPC (16:0), lysoPC (18:0), l-arginine, l-glutamine, l-histidine, l-ornithine, l-serine, and malate.

## 4. Discussion

This study addressed the consequences of three weeks of protein restriction on the plasma and 24-h urine biomarkers of protein deficiency. We used different gradation of protein deficiency, including very low (3% and 5%) for the P3 and P5 groups, respectively; moderately low (8% and 12%) for the P8 and P12 groups, respectively; or adequate (15% and 20%) for the P15 and P20 groups, respectively. The severely and moderate deficient diets (P3, P5, and P8) induced a large decrease in body weight gain, mainly owing to the decrease in the gain of lean mass (results not shown). Metabolomics analyses revealed that protein restriction caused observable perturbation of the plasma and urinary metabolic profiles of rats, corresponding to several adaptive mechanisms.

Firstly, a low protein diet (P3 and P5 of protein) induced a decrease in the concentrations of most EAA in the portal vein (l-lysine, l-methionine, l-phenylalanine, l-threonine, tryptophan, and l-valine), in the vena cava (l-leucine, l-lysine, l-methionine, l-phenylalanine, and l-threonine), and of l-leucine in urine. The portal vein being the reflection of food intake, the low protein diet led to a decrease in EAA [18,19], and the same profile was found in the vena cava. In contrary to the other EAA, l-histidine was increased in the portal vein and vena cava under protein restriction, which is consistent with previous findings [20] showing that serum histidine concentration was inversely proportional to the protein content of the diet. Histidine may be elevated due to a low activity of histidase, an enzyme that catalyzes the deamination of l-histidine to *trans*-urocanic acid under a low protein diet [21], or to an increase in protein catabolism with a higher degradation of proteins containing high concentration of histidine [22]. The elevated creatine in restricted rats’ urine suggested an increase in muscle breakdown. The protein intake does not meet protein needs and muscle proteins were mobilized as indicated, but a decrease in weight gain and lean mass was observed in our study. Other AAs, l-ornithine and l-serine, were increased in the plasma under protein restriction, which can be explained by the increase in protein catabolism. A similar increase in plasma serine was found in rats fed protein- or amino acid-restricted diets [23,24,25]. It was shown that the increase in serine concentration was due to an increase in de novo synthesis with an increase in enzymes involved in serine biosynthesis in the liver [24]. Serine is a precursor of several molecules such as AA, lipids, or ceramides, whose demand could be increased in case of protein restriction.

The present results showed that protein restriction induced an increase in the activity of enzymatic systems of catabolism of certain AAs, leading to degradation metabolites being found in the plasma and urine. Notably, the tryptophan pathway was impacted by protein restriction. Indoxyl sulfate, produced from tryptophan by intestinal bacteria [26], was decreased in the portal vein and urine, and *N*-acetyl-serotonine decreased in the vena cava. It has already been reported that plasma and urine indoxyl sulfate was decreased in response to a threonine deficient diet in the case of caloric restriction [27]. Tryptophan metabolism is connected to nicotinamide metabolism, which is impacted by protein deficiency. It was reported that the excretion of nicotinamide metabolites, as *N*-methyl-2-pyridone-5-carboxamide (2-Py), in the plasma and urine is modified by protein status [28,29]. Rats fed inadequate AA diets have an increased excretion of *N*-methylnicotinamide (MNA) and a decrease in urinary 2-Py and 4-Py (*N*-methyl-4-pyridone-3-carboxamide). In the present study, MNA and 4-Py were found in neither the plasma nor the urine, and the decreases of 2-Py and 4-Py seems to be the result of a decrease in the activity of 2-Py-forming and 4-Py-forming MNA oxidase. The excretion ratio of (2-Py + 4-Py)/MNA has been used as a marker of amino acid adequacy in rats [29]. Thus, the modification of 2-Py in the present study should more likely reflect the protein deficiency.

The lysine degradation pathway was also impacted by the protein deficient diet. l-carnitine and *O*-acetyl-l-carnitine were inversely related to protein content in the diet, while other metabolites such as cadaverine and pipecolate were positively related to protein content in the diet. In addition to the impact of protein restriction on AA metabolism, the enterohepatic cycle of bile acids was modified by protein deficiency. Betaine was decreased when protein increased in the diet in the portal vein and vena cava. Moreover, conjugated bile acids, such as taurocholic acid, were decreased in the portal vein when protein decreased in the diet. Interestingly, in an intervention study on 10 volunteers, a high-fat, high-protein diet increased the plasma concentration of some bile acids (deoxycholic acid, chenodeoxycholic acid, and cholic acid) compared to the control and high-fat, low-protein diets [30]. In contrast, another study on 16 healthy human subjects fed a diet composed of carbohydrates, proteins, or lipids with equal total caloric content reported an increase in total plasma bile acid levels only after a diet composed of lipids compared to the carbohydrate or protein diets [31]. The present study showed that Krebs cycle intermediates were increased with a low protein diet as malate in the plasma and succinic acid in the urine. Other studies based on the effect of protein-energy malnourished rats have shown that there is a decrease in Krebs cycle intermediates. This difference is probably due to the energy deficiency [32,33].

The last group of markers found in the urine correspond to monosaccharides. In the present study, protein deficiency was obtained at the expense of carbohydrates, and the low protein diet was high in carbohydrates compared to the normal protein diet. This increase in carbohydrates in the low protein diet resulted in an increase in carbohydrates in the urine, where different monosaccharides were found, such as glucose, raffinose, galactose, sucrose, mannose, and isomaltose.

The present study showed that AA deficient diets have an important impact on the metabolism of growing rats. Metabolomics analysis provides different biomarkers that could be used to determine protein and AA deficiency. These metabolites could be interesting candidates. The metabolomic profile of the portal vein is very close to that of the vena cava. The same EAA, Krebs cycle intermediate and metabolic products of nicotinamide adenine dinucleotide degradation, were found in the portal vein and vena cava plasma. Only certain AA degradation metabolites differed between the portal vein and vena cava. The comparison of metabolomics profiles between the plasma and urine indicate that most of the metabolites present in the plasma were also found in the urine. Thus, the urinary metabolome seems sufficient to determine biomarkers of AA sufficiency. FAO recommends the use of a non-invasive method to measure AA requirements, and urine collection appears to be a simple approach to implement and a non-invasive method that could be easily used to measure requirements in fragile populations (children and the elderly) or in developing countries where malnutrition is significant.

## Figures and Tables

**Table 1:** Macronutrient composition of the diets. Diets were produced by the “Sciences de l’Animal et de l’Aliment de Jouy (SAAJ), INRAE, Jouy en Josas, France (P stands for Protein).

	P3	P5	P8	P12	P15	P20
Weight content (g/kg)						
Milk proteins	29	48	77	116	145	193.5
Corn Starch	717.9	701.5	676.6	643.1	618.1	576.4
Sucrose	115.8	113.2	109.1	103.6	99.6	92.8
Soy Oil	40	40	40	40	40	40
Minerals	35	35	35	35	35	35
Vitamins	10	10	10	10	10	10
Cellulose	50	50	50	50	50	50
Choline	2.3	2.3	2.3	2.3	2.3	2.3
Energy content (%)						
Protein	3	5	8	12	15	20
Carbohydrate	86.6	84.6	81.6	77.6	74.6	69.5
Fat	9.3	9.3	9.3	9.3	9.3	9.3
Energy density (kJ/g)	14.54	14.55	14.55	14.56	14.56	14.57

**Table 2:** Discriminant urinary metabolites obtained with PLS and ICDA. Data are presented as changes from P3 to P20: ↑ represents an increase and ↓ a decrease. When the changes were not linear among the protein levels, the details are indicated. When no sign was written, the metabolite was not discriminant. For more details, see Figures S1 and S2.

Urinary Discriminant Metabolites	Effect of Dietary Protein Content from P3 to P20	
	PLS	ICDA
3-methyl-2-oxovalerate	↓	↓
4-methyl-2-oxovalerate	↓	↓
oxovaleric acid	↓	↓
azelate	↓	↓
creatine	↓	↓
d-mannose	↓	↓
d-raffinose	↓	↓
galactose	↓	↓
phosphoric acid	↓	↓
pyridoxine	↓	↓
raffinose	↓	↓
succinic acid	↓	↓
pantothenate	↓ P5–P20	↓
alpha d-glucose		↓
galactitol		↓
*iso*-maltose		↓
l-carnitine		↓
proline-leucine		↓
sucrose		↓
anthranilate	↑	↑
cadaverine	↑	↑
homogentisic acid	↑	↑
isovaleroylglycine	↑	↑
kynurenic acid	↑	↑
l-gulonolactone	↑	↑
*n*-methyl-2-pyridone-5carboxamide	↑	↑
pipecolate	↑	↑
uracil	↑	↑
xanthurenate	↑	↑
indoxyl sulfate	↑	↑
putrescine	↑	↑
4-pyridoxate,		↑
l-leucine		↑
*n*-acetylputrescine		↑
spermidine,		↑
tyramine		↓P3–P5↑P8–P12↓P15P20

**Table 3:** Discriminant plasma metabolites obtained with PLS and ICDA in the portal vein (PV) and vena cava (VC). Data are presented as changes from P3 to P20: ↑ represents an increase and ↓ a decrease. When the changes were not linear among the protein levels, the details are indicated. When no sign was written, the metabolite was not discriminant. For more details, see Figures S2 and S3.

Discriminant Plasma Metabolites	PLS		ICDA	
	PV	VC	PV	VC
betaine	↓	↓	↓	↓
fucose	↓	↓	↓	↓
lysoPC (18:0)	↓	↓	↓	↓
l-carnitine	↓		↓	
l-histidine	↓	↓	↓	↓
l-serine	↓	↓	↓	↓
malate	↓	↓		↓
*o*-acetyl-carnitine	↓		↓	
2-hydroxyisocaproic acid		↓	↓	
l-ornithine		↓	↓	↓
pyroglutamate			↓	
l-arginine			↓	↓
citrulline			↓	
l-glutamine				↓
l-methionine	↑	↑	↑	↑
l-phenylalanine	↑	↑	↑	↑
l-threonine	↑	↑	↑	↑
l-tyrosine,	↑	↑	↑	↑
*n*-methyl-2-pyridone-5-carboxamide	↑	↑	↑	↑
taurocholic acid,	↑		↑	
tryptophan	↑		↑	↑
3-isopropylmalic acid		↑		
l-lysine		↑	↑	↑
indoxyl sulfate			↑	
l-valine			↑	
l-leucine				↑
*n*-acetylserotonin				↑

## Supplementary Materials

The following are available online at https://www.mdpi.com/article/10.3390/nu13051567/s1, Table S1: Amino composition of diet. SAA: sulfur amino acid; AAA: aromatic amino acid; Figure S1: Urine (a, portal vein (b) and vena cava (c) metabolites obtained with PLS. Data are presented in mean ± SEM (*n* = 6 per group). ^a, b, c, d^ Data that do not share the same letter are different *p* < 0.05; Figure S2: Urine (a), vena cava (b) and portal vein (c) metabolites obtained with ICDA. Data are presented in mean ± SEM (*n* = 6 per group). ^a, b, c, d^ Data that do not share the same letter are different *p* < 0.05.
