# Supplementary material for: Plasma and Urinary Amino Acid-Derived Catabolites as Potential Biomarkers of Protein and Amino Acid Deficiency in Rats
Source: Nutrients. 2021 May 7;13(5):1567. doi: 10.3390/nu13051567 (PMC8148556; doi:10.3390/nu13051567)

Supplementary files

Table S1. Amino composition of diet. SAA: sulfur amino acid; AAA: aromatic amino acid

	P3	P5	P8	P12	P15	P20
**Weight content (g/kg)**						
Histidine	0.72	1.19	1.91	2.88	3.60	4.80
Isoleucine	1.38	2.28	3.66	5.52	6.90	9.21
Leucine	2.50	4.14	6.65	10.02	12.52	16.71
Lysine	2.15	3.56	5.71	8.61	10.76	14.36
SAA	0.88	1.46	2.34	3.52	4.40	5.88
AAA	2.62	4.34	6.96	10.49	13.11	17.49
Threonine	1.10	1.82	2.91	4.39	5.49	7.32
Tryptophan	0.35	0.57	0.92	1.39	1.73	2.31
Valine	1.67	2.76	4.43	6.67	8.33	11.12
Arginine	0.90	1.49	2.38	3.59	4.49	5.99
Methionine	0.70	1.16	1.85	2.79	3.49	4.66
Phenylalanine	1.27	2.09	3.36	5.06	6.33	8.44
Alanine	0.85	1.41	2.26	3.40	4.25	5.67
Aspartic acid	1.97	3.26	5.22	7.87	9.84	13.13
Cystein	0.18	0.30	0.48	0.73	0.91	1.22
Glutamic acid	5.39	8.92	14.31	21.56	26.95	35.96
Glycine	0.48	0.79	1.26	1.90	2.38	3.18
Proline	2.48	4.11	6.59	9.93	12.42	16.57
Serine	1.38	2.29	3.67	5.53	6.91	9.22
Tyrosine	1.36	2.25	3.61	5.43	6.79	9.06

Figure S1. Urine (a), portal vein (b) and vena cava (c) metabolites obtained with PLS.
Data are presented in mean ± SEM (n = 6 per group). [a, b, c, d] Data that do not share the same letter are different p<0.05.

a. Urine

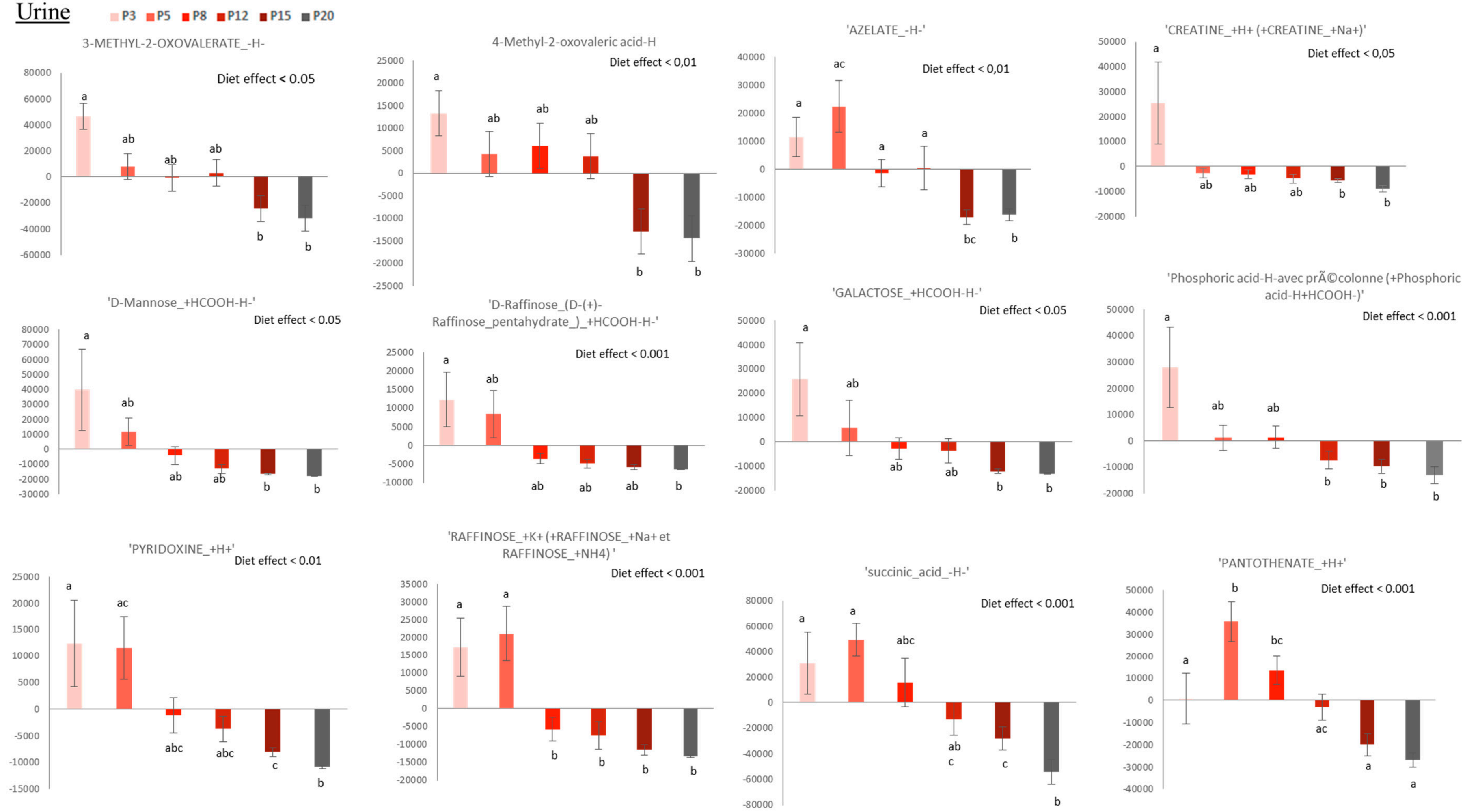

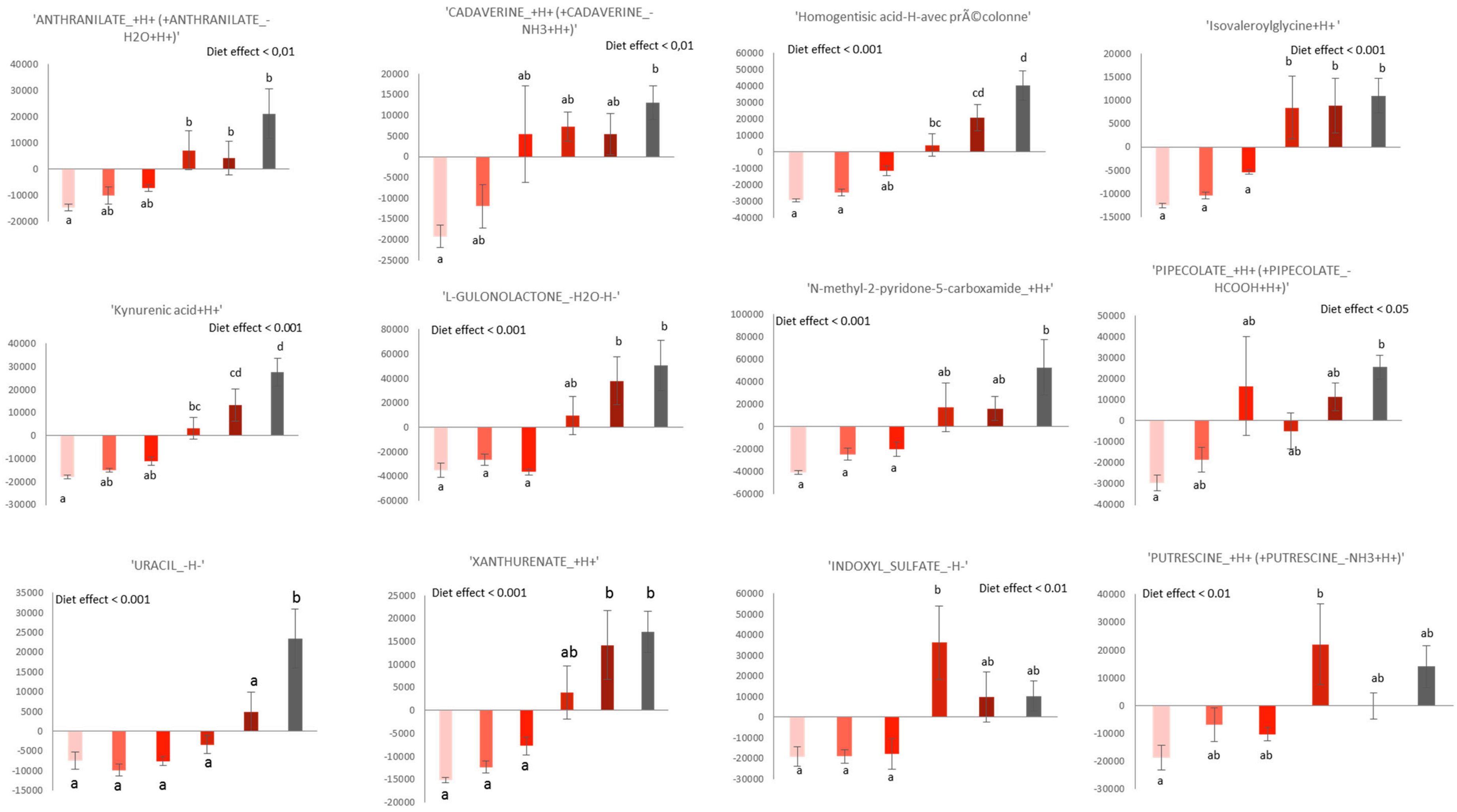
'ANTHRANILATE_+H+ (+ANTHRANILATE_-H2O+H+)'
Diet effect < 0,01
'CADAVERINE_+H+ (+CADAVERINE_-NH3+H+)'
Diet effect < 0,01
'Homogentisic acid-H-avec prÃ©colonne'
Diet effect < 0.001
'Isovaleroylglycine+H+'
Diet effect < 0.001
'Kynurenic acid+H+'
Diet effect < 0.001
'L-GULONOLACTONE_-H2O-H-'
Diet effect < 0.001
'N-methyl-2-pyridone-5-carboxamide_+H+'
Diet effect < 0.001
'PIPECOLATE_+H+ (+PIPECOLATE_-HCOOH+H+)'
Diet effect < 0.05
'URACIL_-H-'
Diet effect < 0.001
'XANTHURENATE_+H+'
Diet effect < 0.001
'INDOXYL_SULFATE_-H-'
Diet effect < 0.01
'PUTRESCINE_+H+ (+PUTRESCINE_-NH3+H+)'
Diet effect < 0.01

b. Plasma vena cava

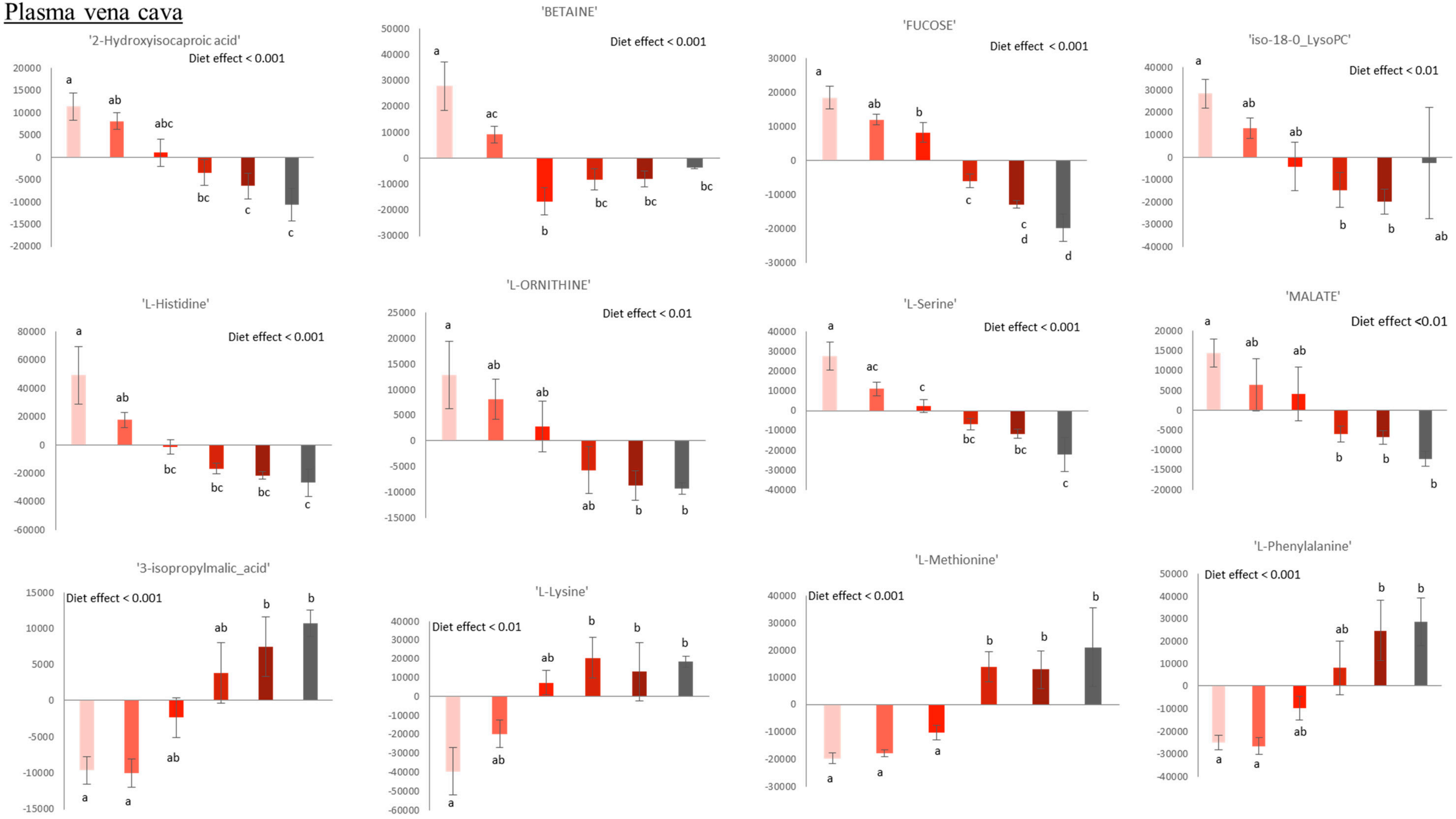

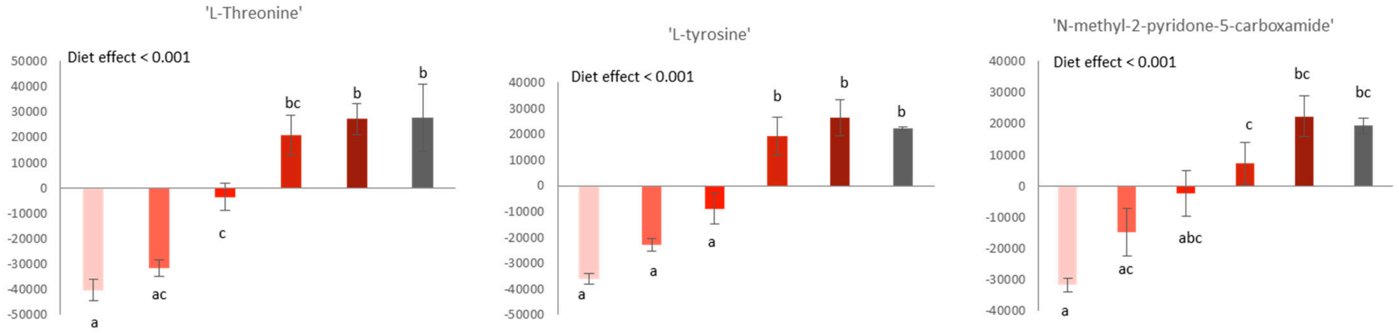

c. Plasma portal vein

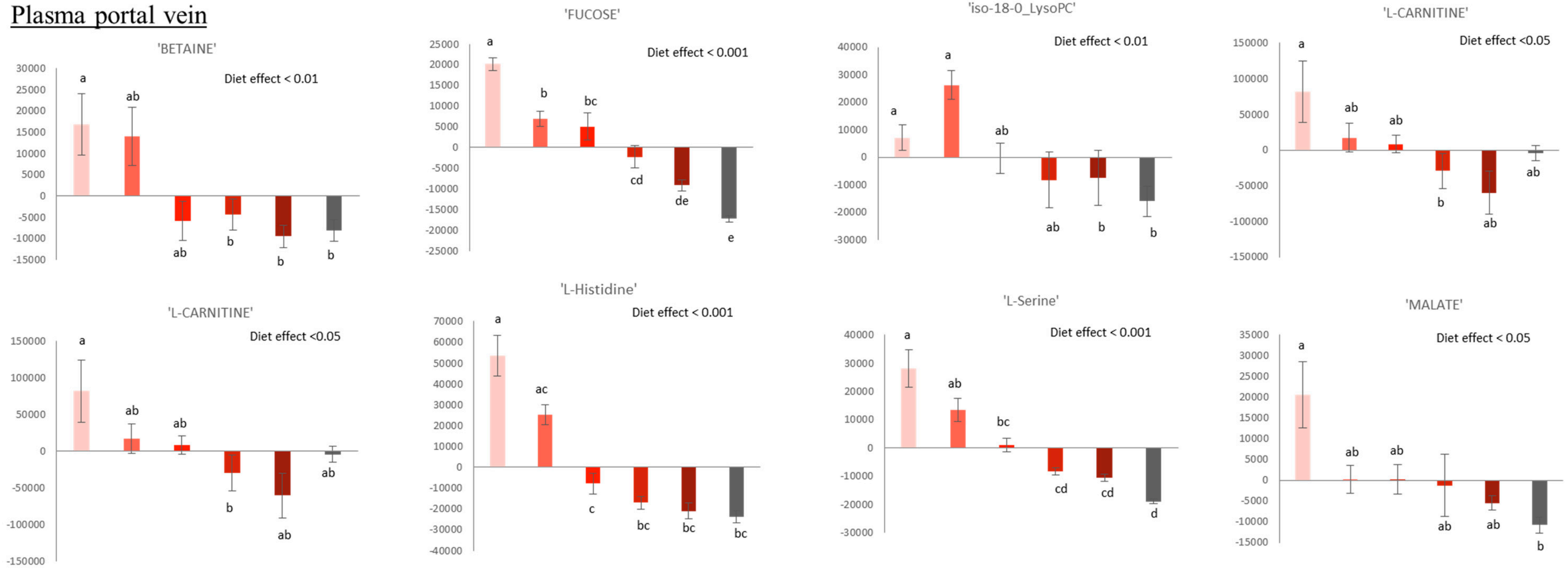

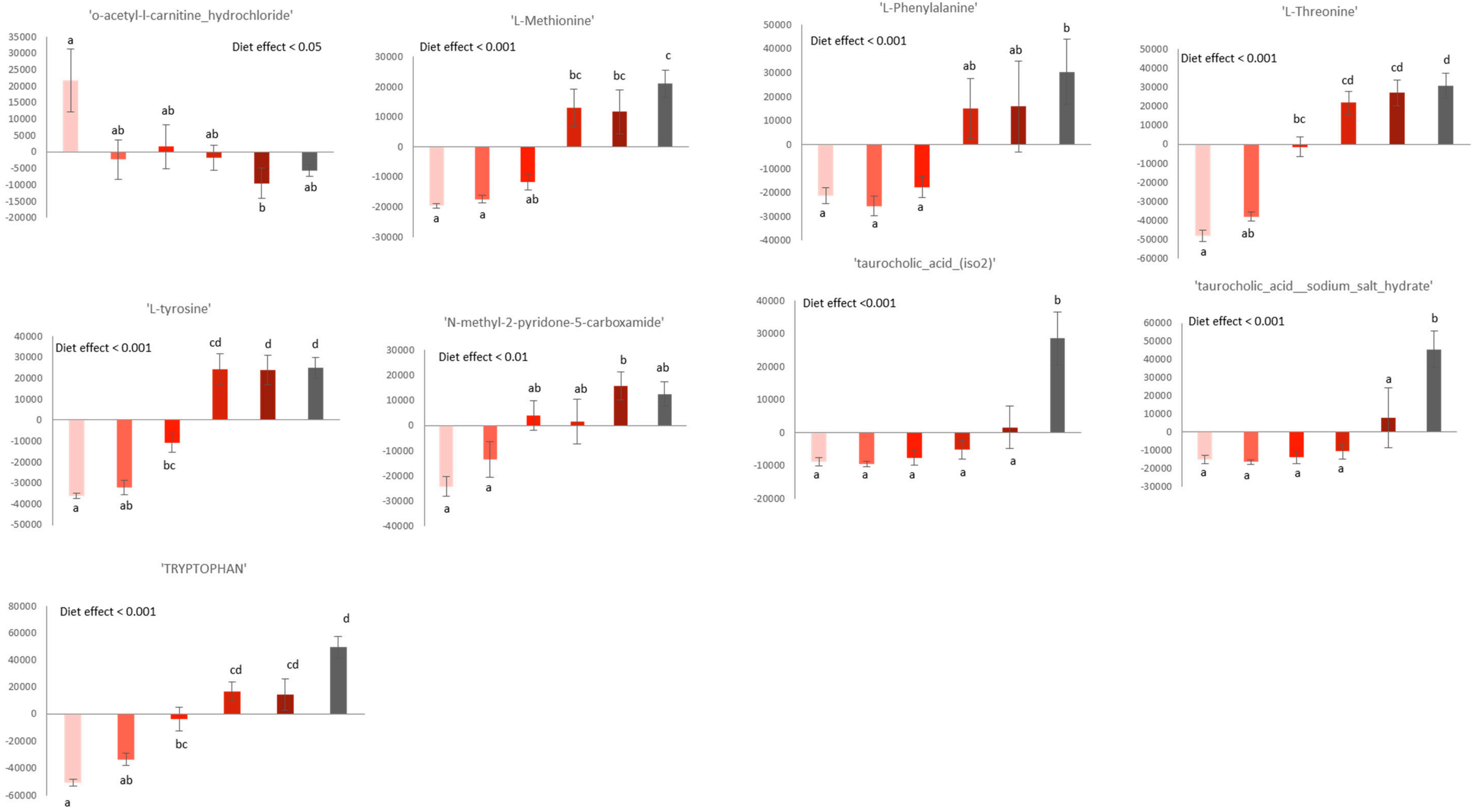
'o-acetyl-l-carnitine_hydrochloride'
Diet effect < 0.05
a ab ab ab b ab
'L-Methionine'
Diet effect < 0.001
a a ab bc bc c
'L-Phenylalanine'
Diet effect < 0.001
a a a ab ab b
'L-Threonine'
Diet effect < 0.001
a ab bc cd cd d
'L-tyrosine'
Diet effect < 0.001
a ab bc cd d d
'N-methyl-2-pyridone-5-carboxamide'
Diet effect < 0.01
a a ab ab b ab
'taurocholic_acid_(iso2)'
Diet effect <0.001
a a a a a b
'taurocholic_acid__sodium_salt_hydrate'
Diet effect < 0.001
a a a a a b
'TRYPTOPHAN'
Diet effect < 0.001
a ab bc cd cd d

Figure S2. Urine (a), vena cava (b) and portal vein (c) metabolites obtained with ICDA.
Data are presented in mean ± SEM (n = 6 per group). [a, b, c, d] Data that do not share the same letter are different p<0.05.

a Urines

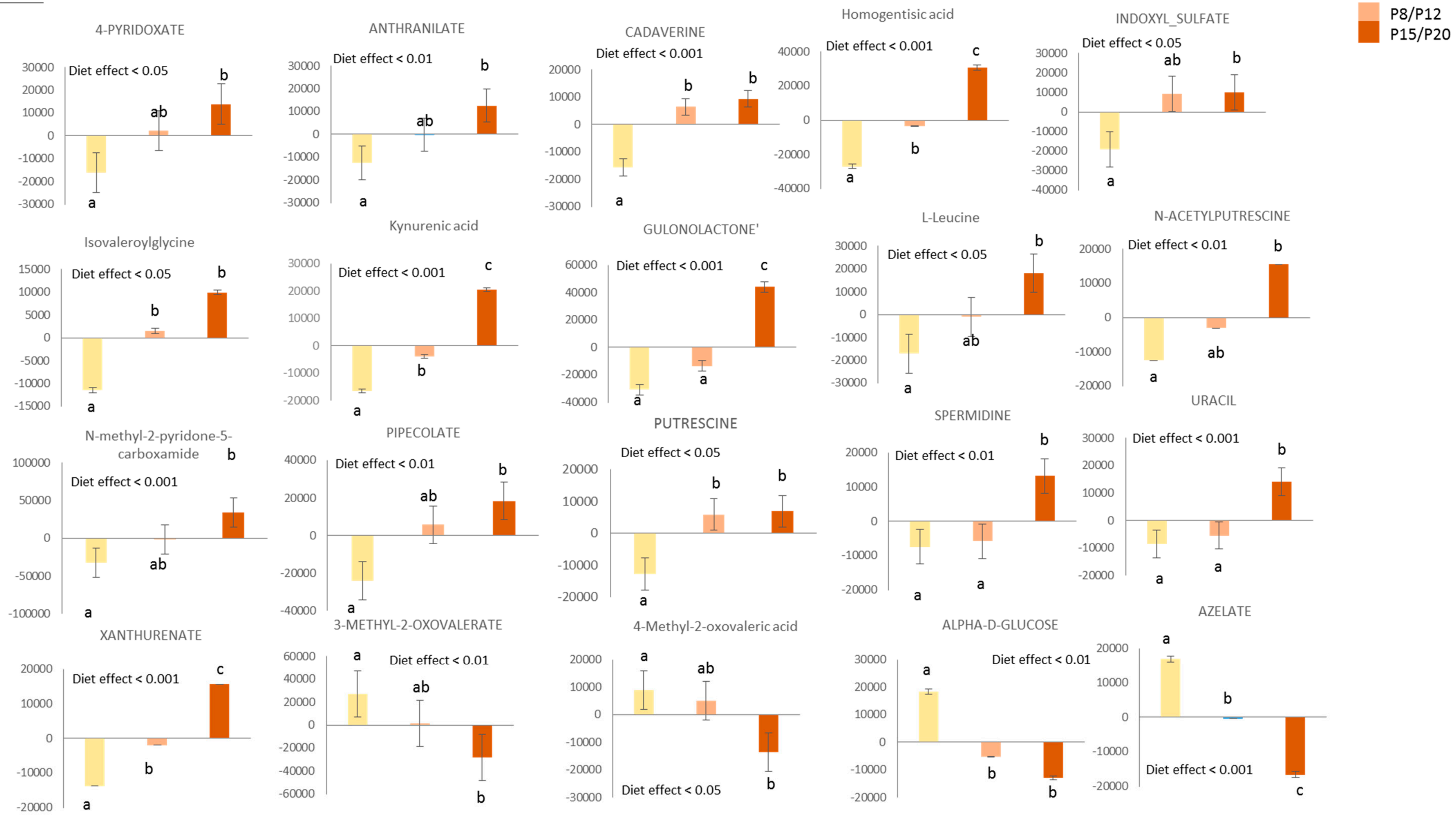

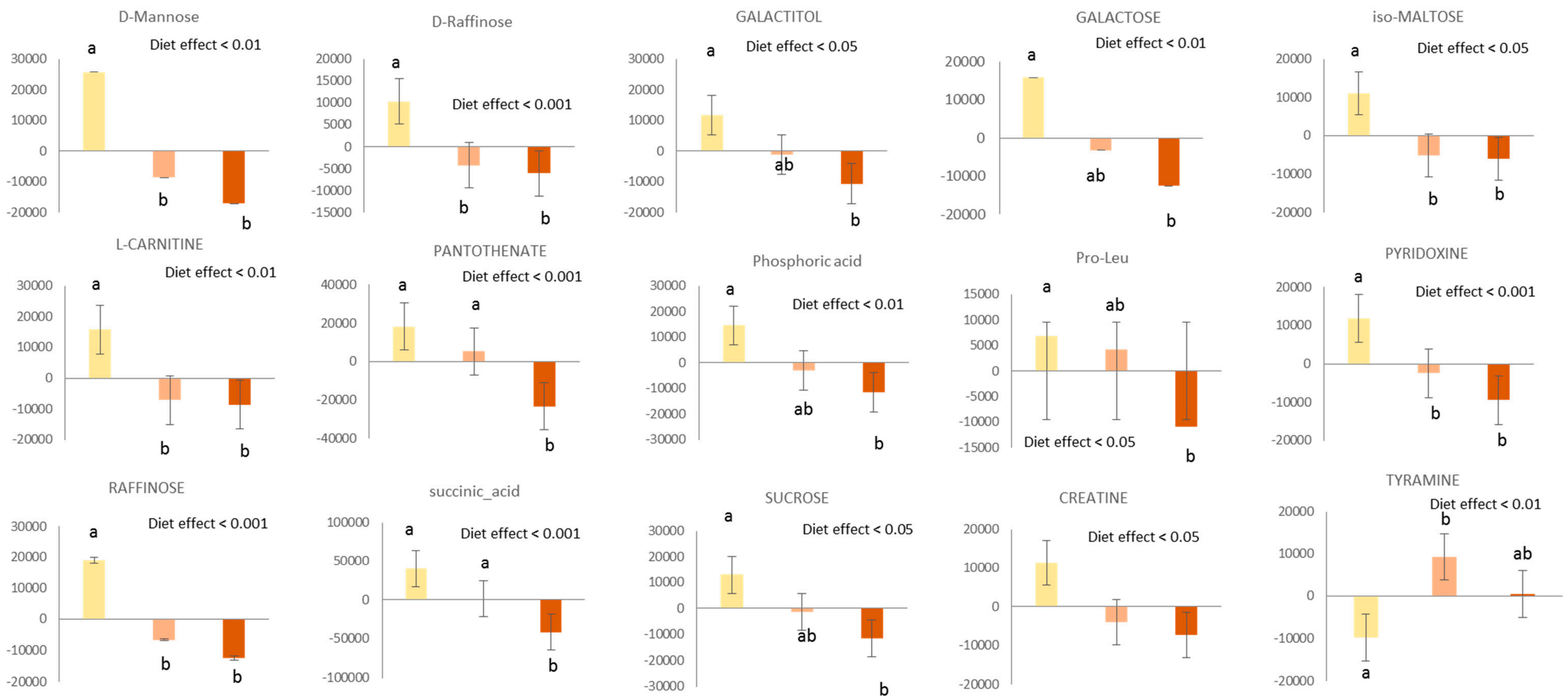
D-Mannose
Diet effect < 0.01
a
b
b
30000
20000
10000
0
-10000
-20000
D-Raffinose
Diet effect < 0.001
a
b
b
20000
15000
10000
5000
0
-5000
-10000
-15000
GALACTITOL
Diet effect < 0.05
a
ab
b
30000
20000
10000
0
-10000
-20000
GALACTOSE
Diet effect < 0.01
a
ab
b
20000
10000
0
-10000
-20000
iso-MALTOSE
Diet effect < 0.05
a
b
b
20000
10000
0
-10000
-20000
L-CARNITINE
Diet effect < 0.01
a
b
b
30000
20000
10000
0
-10000
-20000
PANTOTHENATE
Diet effect < 0.001
a
a
b
40000
20000
0
-20000
-40000
Phosphoric acid
Diet effect < 0.01
a
ab
b
30000
20000
10000
0
-10000
-20000
-30000
Pro-Leu
Diet effect < 0.05
a
ab
b
15000
10000
5000
0
-5000
-10000
-15000
PYRIDOXINE
Diet effect < 0.001
a
b
b
20000
10000
0
-10000
-20000
RAFFINOSE
Diet effect < 0.001
a
b
b
30000
20000
10000
0
-10000
-20000
succinic_acid
Diet effect < 0.001
a
a
b
100000
50000
0
-50000
-100000
SUCROSE
Diet effect < 0.05
a
ab
b
30000
20000
10000
0
-10000
-20000
-30000
CREATINE
Diet effect < 0.05
20000
10000
0
-10000
-20000
TYRAMINE
Diet effect < 0.01
b
ab
a
20000
10000
0
-10000
-20000

b Plasma vena cava

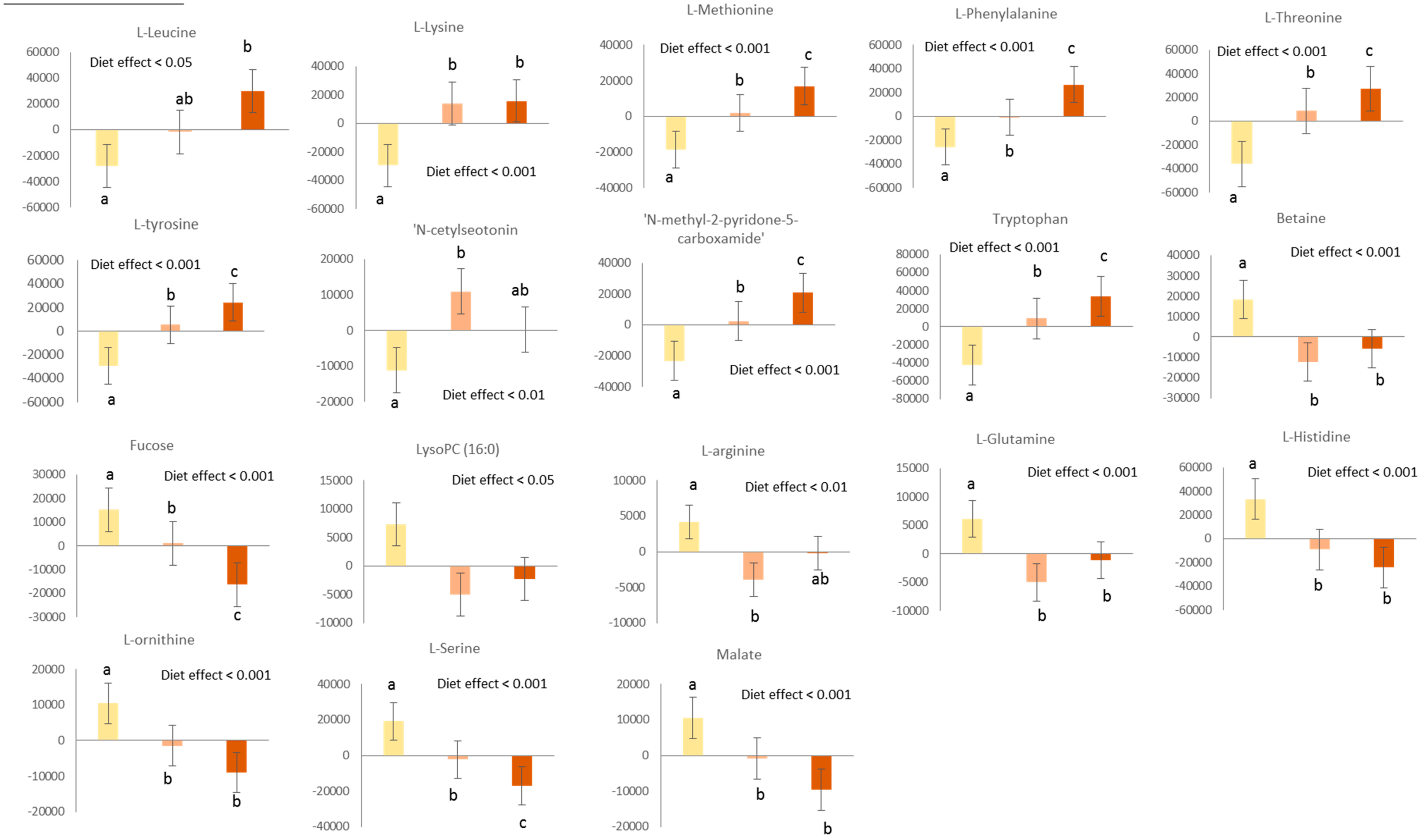

c Plasma portal vein

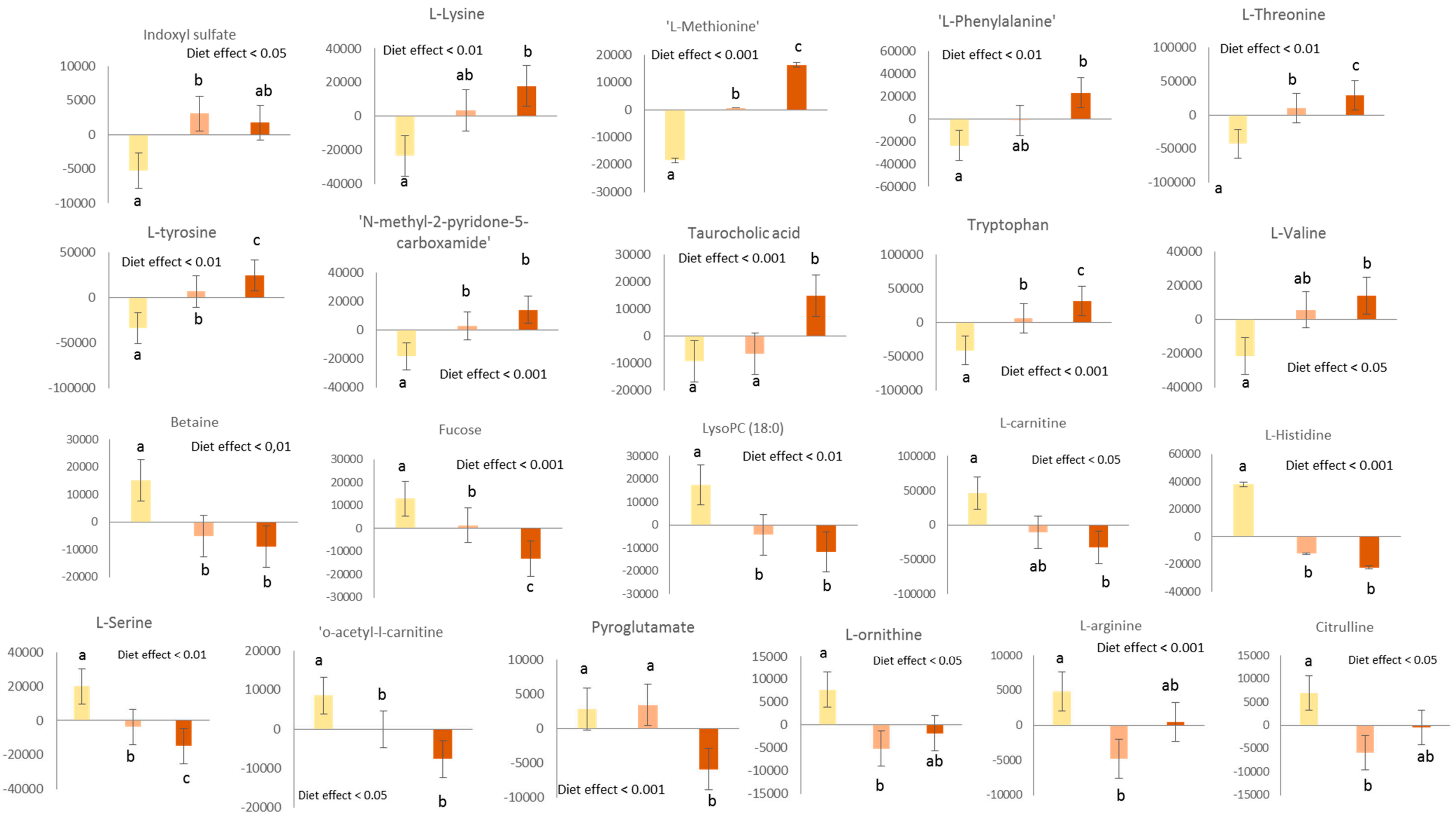

Supplement: Supplementary file 1 [file nutrients-13-01567-s001.zip › nutrients-1184778-supplementary.pdf]
